# Glycans as Potential Diagnostic Markers of Traumatic Brain Injury

**DOI:** 10.3390/brainsci11111480

**Published:** 2021-11-09

**Authors:** Mårten Kvist, Lasse Välimaa, Adrian Harel, Jussi P. Posti, Melissa Rahi, Ilkka Saarenpää, Mikko Visuri, Anna Östberg, Jaakko Rinne

**Affiliations:** 1 Medicortex Finland Oy, 20520 Turku, Finland; lasse.valimaa@medicortex.fi (L.V.); adrian.harel@medicortex.fi (A.H.); 2 Neurocenter, Department of Neurosurgery, Turku University Hospital, University of Turku, 20520 Turku, Finland; jussi.posti@tyks.fi (J.P.P.); melissa.rahi@tyks.fi (M.R.); ilkka.saarenpaa@tyks.fi (I.S.); mikko.visuri@tyks.fi (M.V.); anna.ostberg@tyks.fi (A.Ö.); jaakko.rinne@tyks.fi (J.R.)

**Keywords:** TBI, lectin, glycan, biomarker, concussion, traumatic brain injury, clinical trial, diagnostic test

## Abstract

The diagnosis of mild traumatic brain injury (TBI) is challenging in the acute setting because the symptoms are nonspecific and often transient, or they develop with a delay. In these cases, the criteria for acute head imaging are frequently not fulfilled. This may lead to missed diagnoses in emergency care. There is a need for developing a rapid diagnostic test to verify the presence of TBI using body fluids. Blood, urine, and saliva samples from 11 adult patients (mean age 64 years, SD 24 years) with acute and clinically diagnosed TBI, and 12 healthy volunteers were collected at Turku University Hospital during a period of 5 months. The injuries necessitated hospitalization for at least one day. The TBIs were classified mild in nine cases and severe in two cases. The mean period between the trauma and the time for obtaining the samples was 27 h, SD 11 h. The samples were analyzed in an ISO-certified laboratory for the number of lectin-bound glycan molecules indicating destruction of nerve tissue. The screening was performed on several possible glycans for binding, and the measurement by degree of fluorescence. In the analysis, the group of patients with TBI was compared with healthy volunteers. The results showed a significant decrease (*p* < 0.05, Wilcoxon rank–sum two-sided test) in the level of two glycans in *plasma*, but no significant increase for any glycan; in *saliva*, one glycan showed a significant increase in the TBI group; in *urine*, three glycans were significantly different between the groups (one showed an increase, whereas two showed a decrease). The results support the idea of conducting more research on how diagnostic glycans could be detected in body fluids after TBI. As a proof-of-concept, significant changes in the concentration of five glycans were found in plasma, saliva, and urine between TBI patients and healthy controls. This may enable the development of a rapid body fluid-based point-of-care test to identify patients with TBI after a head injury.

## 1. Introduction

Traumatic brain injury is caused by an impact on the head or by a rapid movement of the head leading to altered brain function. Mild traumatic brain injury (mTBI) and concussion are particularly difficult to diagnose [1,2,3]. 

Computed tomography (CT) is used to detect traumatic intracranial findings in patients with head injuries, but it cannot be used to exclude TBI. Besides CT, some biomarkers have been claimed to detect brain injuries, e.g., S100β, which was introduced more than 50 years ago [4]. More recently, a study assessed whether S-100β protein could be measured in urine when detectable in plasma after a mTBI. However, urine sampling did not appear to be useful during the acute phase after mTBI [5]. Furthermore, an FDA-approved glial fibrillary acidic protein and ubiquitin carboxy-terminal hydrolase L1 (GFAP-UCH-L1) test to detect intracranial abnormalities [6] is currently in use in Scandinavian countries, and it has recently been validated in the Finnish population [7]. Several other biomarkers to detect mTBI have been developed over the past years, but none of them have been found to be ideal and have the potential to replace a clinical evaluation and CT scan. Review articles of available biomarkers for TBI have been published recently by Carney et al. and Harel et al. [8,9]. 

This study was conducted to collect clinical samples for the development of a biomarker, preferably using either saliva or urine instead of blood for testing, that could be used in clinical practice. The study intended to create a proof-of-concept for the development of a rapid biochemical diagnostic kit for TBI detection. A new rapid diagnostic test based on a new biomarker would significantly improve the early detection of a brain injury and would help in the treatment stratification of patients with a head injury [10,11]. 

The hypothesis for this research was that glycans may function as biomarkers for detecting brain injury.

## 2. Material and Methods

### 2.1. Study Design

We conducted a prospective study of a small group of patients with acute TBI, and compared their body fluid samples with healthy controls in order to investigate possible differences in their glycan concentration and composition using lectin-binding methods.

The Ethics Committee of the Hospital District of Southwest Finland approved the project on 28 July 2016 (T129/2016). The study was registered on 19 July 2016 in clinical trials with the registration number NCT02836951. The clinical phase of the study—registration of the patients and collection of the body fluid samples—was conducted between 13 September 2016 and 31 January 2017.

### 2.2. Settings and Population

Eleven patients who had suffered an impact on the head and had been hospitalized at Turku University Hospital because of a TBI suspicion were recruited. After the patients had given their informed consent to the study, samples were taken.

The patients, who had a head trauma and had been treated in the hospital ward because of a clinically diagnosed TBI, were classified as TBI patients, whereas the control group consisted of healthy volunteers who were not aware of any head trauma during the previous 3 months. The classification of the severity of the TBI followed the Finnish treatment guidelines for TBI [1]. Patients who had a Glasgow coma score of 13–15, including even slight changes in a CT scan that were TBI-related, were considered to have mild TBI; patients with a Glasgow coma score between 8–12 and showed signs of TBI in a CT scan were considered to have moderate TBI; and patients with a Glasgow coma score with a maximum of 8 and showed signs of TBI in a CT scan were considered to have severe TBI.

From the suspected TBI patients, 2 mL of blood, 2 mL of saliva, and 2 mL of urine were collected. Blood samples were withdrawn using a venous puncture as a normal part of the hospital treatment. The blood samples were collected into a sodium citrate vacuum tube and centrifuged, and only the separated plasma was taken into storage. Before the saliva samples were collected, the patients had to rinse their mouth twice with pure water. Thereafter, they spit saliva into a clean plastic cup. At least one hour must have elapsed since last eating. The urine samples were collected in a plastic cup and transferred to storage vials. 

All specimens were moved as soon as possible to a freezer with a temperature of −70 °C, and stored (up to a few months) until analyzed together in the laboratory. Because of the work safety requirement of the biochemical laboratory, all study subjects were also tested for HIV and hepatitis B, but all tests were negative.

The study subjects were monitored for any symptoms or side effects caused by sampling procedures. If major harmful effects (leakage, unexpected pain) occurred due to the samplings, the subject could finish their participation in the study.

Patients with suspected TBI underwent head CT scans according to the NICE (National Institute for Health and Care Excellence) criteria [3]. All patients were treated according to the local protocols based on existing international guidelines and recommendations at that time [1].

The samples from healthy controls were taken as agreed and scheduled between the subject and the study coordinator.

All patients were above 18 years of age. In addition, the 12 subjects who had not suffered any head trauma (self-reported) in the past three months, were included as healthy controls, and they gave the same equivalent samples as the injured patients. This group consisted mainly of staff members of Turku University Hospital.

### 2.3. Inclusion Criteria

The inclusion criteria were:

(1) Patient was hospitalized because of a head trauma and a suspected TBI.
(2) Patient was aged 18 or above. The subject (including the control subjects) had to be an independent adult who was not under guardianship.
(3) The patient (including the control subjects), or the next of kin in the case of an incompetent person, had to sign the informed consent.
(4) The samples could be collected during regular office hours.

### 2.4. Exclusion Criteria

The exclusion criteria were:

(1) The subject was found positive for HIV or hepatitis B in the involved laboratory studies.
(2) The subject had a chronic mental disorder which was mentioned in the patient records.

The physician in charge of the treatment of the TBI patient in the hospital ward recorded the final diagnosis based on his overall clinical assessment at discharge of the patient. This included a radiologist’s statement of the possible findings in a CT scan, measurement of the Glasgow coma scores, and a clinical examination. Only patients with a final diagnosis of TBI were included in the TBI group.

During the study period, one monitoring visit was paid by the sponsor to secure that data were collected and recorded according to the protocol.

### 2.5. Biochemical Analysis Procedures

Glycans have an affinity to bind to lectins. They have high binding specificity for their target glycan structure and a moderate binding strength (affinity).

For identifying the glycan compositions (potential TBI biomarkers), the samples were analyzed in an ISO-certified laboratory in France (Tebu-Bio Ltd., Le Perray en Yvelines, France). The biochemical studies were performed in two batches; the first one when samples of nine patients and 12 controls were collected, and the rest were analyzed when all the remaining samples had been collected. The samples were shipped frozen under controlled conditions and by qualified parcel services to the receiving analysis laboratory. The laboratory performed the biochemical measurements blinded, without knowing which study group the sample belonged to. The category was disclosed to the service provider only after the laboratory data were retrieved. The research laboratory received only the minimum clinical background details which were relevant for the analysis.

The samples were studied by a biochemical glycan-binding analysis [12]. In brief, the procedure as follows: The sample was incubated with immobilized lectins arrayed on a glass slide in order to capture the glycans of interest from the sample. The excess fluid and unbound material were washed away, and the bound biomarkers were visualized by fluorescent reporter molecules. For discovering the glycans in the clinical samples, we used up to 70 lectins representing multiple, different glycan specificities (Lectin Array 70, RayBiotech^®^, Peachtree Corners, GA, USA) The measured fluorescence intensities were proportional to the amount of the glycan complexes present in the sample. The levels and intensities were analyzed and compared between the study groups.

The biochemical studies were performed in two batches (eight patients and 12 controls in the first batch, and three patients in the second batch). Inter-run normalization was performed between the batches through an internal control sample analyzed in both batches. 

Statistical analyses of the results were conducted using Wilcoxon’s two-tailed rank-sum non-parametric test for comparisons between the TBI group and the healthy control subjects. *p*-values less than 0.05 were considered statistically significant. The Kolmogorov–Smirnov test was used for testing the existence of normality of the distribution, and a fold change was calculated indicating the relationship of the mean values between the TBI group and the controls. 

## 3. Results

The age and sex distribution of the TBI patients and healthy controls is presented in Table 1.

Most of the healthy controls were younger than the TBI patients. None of the healthy controls were part of the oldest age group (65–94 yrs.). Among the healthy controls, the majority were women, whereas there was a predominance of men among the TBI patients.

The average time which had passed since the injury and the sample collection was 27 h 20 min (SD 11 h 30 min).

### 3.1. Clinical Findings

Of the eleven patients treated in the hospital because of TBI, nine had mild TBI and two had severe TBI. The patients were treated according to existing guidelines [1,3]. However, all were conscious when recruited.

For the patients with severe TBI, the Glasgow coma scores were initially 9 or 10, but dropped later to 5 or 6, whereas for the patients with mild TBI, four had a Glasgow coma score of 14 and five had 15.

For all TBI patients, the ICD code for the clinical diagnosis was a subcode of S06 (Table 2). 

Nine out of the eleven patients had one or more concomitant chronic diseases. Five of the eleven patients had other trauma diagnoses as well.

Six out of the eleven patients suffered from hypertension and three had hypothyroidism. Among the patients, 11 other single diagnoses were also recorded. Three patients had no chronic diseases. The comorbidities are listed in Table 3.

A CT scan was performed for all eleven patients. Six out of the eleven patients treated in the hospital initially showed objective findings of TBI in the CT scan, whereas an MRI was additionally performed for one patient who had four days earlier been examined and found to have a negative CT scan result. The MRI showed that even this patient had findings attributable to TBI. Based on the lectin-finding specificities, we found five glycan species that were significantly different in TBI patients compared with healthy controls in at least one of the body fluids. The results are shown in Table 4.

**Figure 1:**
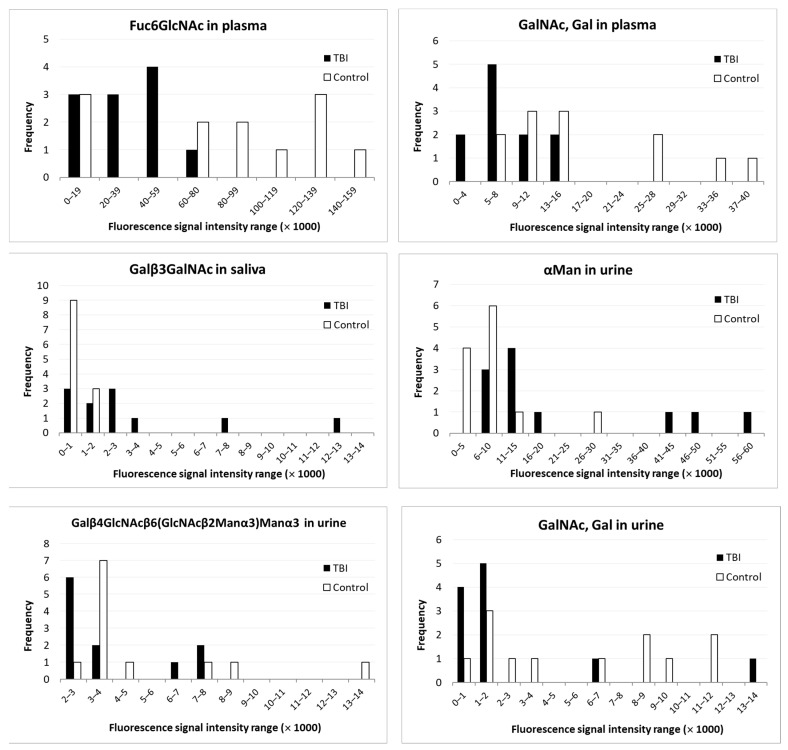
The histograms of glycans which significantly differed between TBI patients and the controls.

Histograms showing the distribution of the glycan measurements among TBI patients and healthy controls are shown in Figure 1. All glycans in the figure showed statistically significant differences between TBI patients and controls.

Compared with healthy controls, in *plasma*, two glycans showed decreased values in TBI patients, and none showed an increased value; in *saliva*, one glycan in TBI patients showed an increased value; and in *urine*, another glycan showed an increased value, whereas two glycans showed decreased values (Table 4).

No glycan showed an increase or decrease in all body fluids. 

### 3.2. Correlation between Clinical Findings and Glycan Analysis Results

Seven out of the eleven TBI patients showed objective findings (either CT scan or MRI) indicative of TBI. Six out of the eleven TBI patients had significantly increased biomarker (Galβ3GalNAc) values in the saliva. Three out of the eleven patients showed significant biomarker (α-mannose) values in the urine. 

Two patients who had a severe TBI had varying results in the glycan analysis of urine. The unit for measurement was the fluorescence intensity measured. For one patient, α-mannose was 5.91 times higher (measured value: 49,502 units) than the mean for the controls (8383 units, SD 6856 units), whereas the other severe TBI patient had normal levels (8227 units). Both patients with severe TBI had signs of TBI in the CT. On the other hand, one TBI patient, who did not show any changes in the CT scan, was later shown to have remarkably high levels of one glycan (GalNac, Gal) in urine: 13,268 units, i.e., 2.38 times higher than the mean value in the control group (5567 units, SD 4260 units).

In addition, one study subject in the control group had surprisingly high levels of one glycan (Galβ4GlcNAcβ6(GlcNAcβα2Man3)Manα3) in the urine (13,414 units, i.e., 2.60 times higher than the mean of the control group’s 5149 units, SD 3131 units) without being aware of any previous TBI.

## 4. Discussion

Glycans are a part of glycoproteins which are released from nerve tissue in the brain when a patient has incurred TBI, and the blood–brain barrier has been damaged. The amount of glycans can be measured if they are bound to lectins, which are derived from plants. Different lectins have different affinities for different glycans. 

The null hypothesis for this study was that there would be no differences in the glycan concentrations in any of the three tested body fluids (plasma, saliva, and urine) between patients with acute TBI in comparison to healthy subjects. As a result of the analysis of the glycan content in body fluids, significant changes were found in all three body fluids. A significant increase for one glycan was found in saliva and for another glycan in urine. Both in plasma and in urine, two glycans were found to have lower concentrations in TBI patients than in controls. Thus, the null hypothesis could be rejected.

The diagnosis of mild TBI is troublesome and debated [1,2]. In the cases of moderate or severe TBI, the clinical diagnosis is less difficult. It has been debated whether mild TBI may show signs of abnormal CT scan results or not. According to the current national guidelines, a small amount of bleeding in the subarachnoid area or a small subdural hematoma may still qualify as mild TBI when the duration of the loss of consciousness and posttraumatic amnesia are short [13].

In this study, the diagnosis of TBI was performed by the physician who was responsible for the treatment of the patients in the ward. It was based both on clinical evidence and the results of CT imaging. A positive result from a CT scan is always evidence of TBI, even if the Glasgow coma scores are 13–15 [14]. If a CT scan does not show any signs of acute TBI, additional evidence may be obtained by measuring the biomarkers for TBI. 

A couple of biomarkers have shown promise for the detection of TBI [6,8,9]. The only biomarker which has been recommended by the Scandinavian Guidelines for the diagnosis of head injuries is S100β [2]. Unfortunately, other injuries may also influence the level of this biomarker; therefore, it is less specific [15]. In 2018, the FDA authorized the first blood test to aid in the evaluation of concussions in adults [16].

Most of the biomarkers studied focus on measuring proteins in the blood or plasma. In this study, the focus was to study the changes in the glycan components of patients with acute TBI. The aim was also to develop a biomarker that could use body fluids other than blood or plasma, and which could also be used in an ambulatory setting. Based on the results of this study, both saliva and urine suggested an increased response for single glycans in patients with TBI. For the development of a simple detection method for the biomarker, an increase in the glycan level is easier to manage than a decrease. The strongest change was observed in saliva (Table 4).

The use of a valid and reliable biomarker may be a valuable addendum to the diagnostic process of TBI in addition to clinical evaluation and imaging diagnostics. The hazards of radiation exposure have been reported by Brenner and Hall [17].

In this study, the patient group was selected amongst individuals treated in a hospital ward because of diagnosed acute TBI. Hospital staff members formed the healthy control group. Surprisingly, among the healthy controls, there was one study subject who showed significant changes in glycan levels without being aware of any previous TBI. A possible explanation could be that the subject had earlier suffered a mild TBI, but was not aware of the symptoms anymore at recruitment into the study, and regarded herself as healthy. For a better interpretation of the results, it would be necessary for the group of patients to be, in addition to broader, more homogeneous, without patients with severe TBI, and matching age, sex, and comorbidities between patients and controls, because these factors may influence the results.

The hospitalized patients were predominantly men. This is in accordance with the findings of other studies [18]. Among the TBI patients in this study, there was also a predominance of elderly people, which is in line with the changing epidemiological pattern of TBI patients in Finland [19]. Among healthy controls, working-aged people were overrepresented. No elderly people were among the controls. The study groups were not age matched.

Of the patients, nine out of eleven had mild TBI. This is in accordance with earlier studies, where about 71–98% of TBI patients have TBI which is classified as mild [18].

Of the eleven patients hospitalized because of TBI, seven showed TBI-induced changes in the imaging of the brain (either CT or MRI). That means a total of 64% of positive CT findings among patients with TBI. In other studies, the prevalence of positive findings has varied between 4.7% to 19% in TBI patients who have had a CT scan of the brain [8]. The difference can be attributed to the small number of cases in this study. The number of patients in this study was small, and it’s premature to conclude whether this test could help decision making in the case of mild TBI. The objective of the study was designed to define whether concentrations of glycans were altered because of a brain injury. The study is of sufficient interest to continue by including more patients with mTBI only and CT or MRI with obvious traumatic lesions.

A significant increase of some glycans was observed in saliva and urine, which implies that a rapid and portable diagnostic kit for the detection of TBI may be possible. The use of a rapid test to detect TBI offers some advantages [3]. First, it facilitates decision making when the test results are easily available with a low cost. It gives additional value and strengthens the validity of the clinical assessment. If the test is sensitive enough, it may diminish the need for a head CT scan. Such examinations are only available in big clinical centers and may require time-consuming travel and costly procedures. Initial testing would also help to define the cases of mTBI, where the patient later has symptoms which can be related to TBI, but an initial clinical interpretation and negative CT imaging result concluded that there was no TBI, e.g., diffuse axonal injury (DAI). It was obvious that the measurements varied considerably between the individuals. The study group, however, was small, and further assessment of the reasons for the variance was not possible. The distribution was not normal for six glycan measurements, as shown by the Kolmogorov–Smirnov test. However, between the groups, the results were convincing, as five glycans showed significant differences.

## 5. Conclusions

This study had enough samples from both TBI patients and healthy controls to proceed with the development of the biomarkers for rapid diagnostics of TBI. As a proof-of-concept, significant changes in the concentration of five glycans were found in plasma, in saliva, and in urine between TBI patients and healthy controls. 

## Figures and Tables

**Table 1:** The distribution of the study subjects according to age group and sex.

	TBI Patients			Healthy Controls			All
Age Group (yrs.)	Males	Females	Total	Males	Females	Total	
15–34	-	2	2	1	4	5	7
35–64	3	-	3	1	6	7	10
65–94	4	2	6	-	-	-	6
Total	7	4	11	2	10	12	23
Age range (yrs.)			21–90			28–61	

Abbreviations used: TBI = traumatic brain injury.

**Table 2:** The distribution of head injury-related ICD-10 codes among the patients with TBI.

Main Diagnosis (ICD-10 Code)	Type of TBI	n
S06.0	Concussion	4
S06.3	Localized brain injury	4
S06.5	Traumatic subdural hematoma	2
S06.6	Traumatic subarachnoid hemorrhage	1

Abbreviations used: TBI = traumatic brain injury; ICD = international classification of diseases.

**Table 3:** The comorbidities in the group of patients with TBI.

ICD-10 Code	n *	Comorbidity
I10	6	Essential hypertension
E03, E03.9	2	Hypothyroidism
E78.1, E78.5	2	Lipoprotein metabolism disturbance
K57.3	2	Diverticulosis of the colon
E11.9	1	Adult type diabetes mellitus
C88.0	1	Malignant immunoproliferative disease
J45.9	1	Bronchial asthma
C17.9	1	Cancer of small intestine
N40	1	Prostate enlargement
J44.8	1	Chronic obstructive pulmonary disease
G20	1	Morbus Parkinson
I25.1	1	Coronary heart disease
-	3	No disease

* up to four comorbidities were recorded per patient.

**Table 4:** Fluorescence measurement intensities in the different body fluids of the 23 study subjects grouped as patients with TBI and uninjured healthy controls. The findings indicate that the concentration of two glycans in plasma were significantly decreased; in saliva and in urine, one glycan was significantly increased, whereas two glycans were significantly decreased in urine.

Glycan	TBI			Healthy					
	Mean	SD	Median	Mean	SD	Median	Fold Change	Wilcoxon *p*-Value	Kolmogorov Smirnov *p*-Value
**Plasma:**									
Fucα6GlcNAc	36,051	19,552	36,893	86,459	48,231	93,698	0.42	0.009	0.003
αMan	39,776	21,877	47,019	50,679	9923	48,794	0.78	N.S.	-
Galβ3GalNAc	15,747	3599	17,300	14,436	3132	14,453	1.09	N.S.	-
Galβ4GlcNAcβ6(GlcNAcβ2Manα3)Manα3	64,897	35,387	52,921	71,915	35,936	63,518	0.90	N.S.	-
GalNAc, Gal	7791	3861	7224	17,657	10,617	14,286	0.44	0.007	0.015
**Saliva:**									
Fucα6GlcNAc	510,391	426,179	381,201	586,647	328,204	503,917	0.87	N.S.	-
αMan	21,472	19,645	12,676	15,764	10,919	14,628	1.36	N.S.	-
Galβ3GalNAc	3280	3779	2059	807	366	910	4.06	0.009	0.060
Galβ4GlcNAcβ6(GlcNAcβ2Manα3)Manα3	4679	2756	3565	4611	2925	4321	1.01	N.S.	-
GalNAc, Gal	14,802	33,932	4005	45,161	52,213	28,409	0.33	N.S.	-
**Urine:**									
Fucα6GlcNAc	38,439	27,672	29,684	41,444	50,090	26,755	0.93	N.S.	-
αMan	21,186	18,747	12,586	8383	6857	5730	2.53	0.006	0.017
Galβ3GalNAc	2580	1873	2354	2044	1225	2435	1.26	N.S.	-
Galβ4GlcNAcβ6(GlcNAcβ2Manα3)Manα3	3763	2110	2549	5149	3132	3791	0.73	0.044	0.066
GalNAc, Gal	2635	3990	1125	5567	4260	5188	0.47	0.027	0.054

N.S. = not significant; TBI = traumatic brain injury. The groups were tested with the Kolmogorov–Smirnov test, and the results indicated that the samples for three of the five glycans were not normally distributed; therefore, the Wilcoxon’s rank-sum two-sided test was used instead of the t-test in the statistical calculations.

## Data Availability

Original patient data stored at the Department of Neurosurgery of Turku University Hospital (TYKS), Turku, Finland. Laboratory data stored at Medicortex Finland Oy, Itäinen Pitkäkatu 4 B, 20520 Turku, Finland. Documentation of the clinical trial: https://clinicaltrials.gov/ct2/show/NCT02836951?term=Medicortex&draw=2&rank=3 (accessed 8 November 2021). Scientific report: Glycans as potential diagnostic markers of traumatic brain injury. Brain Sciences 2022:1:XXX-XXX. Submitted for publication.

## Abbreviations

CT: computed tomography; DAI: diffuse axonal injury; Fuc: fucose; Gal: d-galactose; GalNAc: N-acetylgalactosamine; Glc: d-glucose; GlcNAc: N-scetylglucosamine; HIV: human immunodeficiency virus; IEC: independent ethics committee; Lac: lactose; αMan: α-mannose; MRI: magnetic resonance imaging; mTBI: mild traumatic brain injury; TBI: traumatic brain injury; TYKS: Turku University Hospital; VSSHP: Hospital District of Southwest Finland.
